# Optical waves in a gradient negative-index lens of a half-infinite length

**DOI:** 10.1038/srep02954

**Published:** 2013-10-16

**Authors:** Yi S. Ding, C. T. Chan, R. P. Wang

**Affiliations:** 1 School of physics, Peking University and Department of Physics, The Hong Kong University of Science and Technology; 2 Department of Physics, The Hong Kong University of Science and Technology; 3 School of physics, Peking University

## Abstract

Materials with negative permittivity and permeability can overcome the diffraction limit, thereby making the sub-wavelength imaging possible. In this study, we analyze the effects of gradient index on a half-infinite perfect lens. We assume that the sharp interface between the vacuum and the negative-index material is replaced by a smooth transition profile such that the index gradually changing from positive to negative. Interestingly, we find that if the graded index profile is modeled by a tanh function, we can have closed-form analytical solutions for this problem, which is a distinct advantage as numerical solutions are not accurate for evanescent waves with large transverse wave vectors. By analyzing the analytical formulas we confirm that a nonzero total absorption can occur even for a near-zero absorption coefficient in the steady-state limit and the image plane contains multiple sub-wavelength images of an object.

Pendry^1^ proposed that a slab with 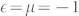 can overcome the diffraction limit leading to the perfect lens concept. The mechanism is that the perfect lens can amplify the evanescent waves and restore the high-spatial-frequency information of an object. The effects of absorption on perfect imaging of a finite-length slab were adequately considered^2^,^3^. Several experimental demonstrations on the subwavelength imaging system^4^,^5^ have been achieved.

In practice, a negative-index material cannot have an infinitely sharp interface between the material and vacuum. From an effective medium point of view, the transition from vacuum to negative index should be a smooth function. In that sense, the perfect lenses can be modeled as positive-to-negative transition materials^6^. As such, they have been modeled as layered optical materials with gradient optical indexes 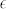 and/or *μ* continuously changing from positive values to negative ones. In Ref. 7 and 8, it is found that near the transition point for 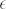, the fields for oblique incidence present large enhancement with enhanced absorption. The absorption is found to be nonzero even in the lossless limit. In Ref. 6, similar effects are obtained when both 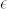 and *μ* linearly pass the same transition point. The question we are addressing here is the effect of the zero-index point on the quality of perfect imaging.

In order to answer that question we consider a simplified case, a half infinite negative-index material with a gradient index profile described by the function tanh. For simplicity, that material is free of frequency dispersion. To study the imaging effects, we need to analyze the whole spectrum of the object. However, numerical methods (such as transfer matrix method) for evanescent waves can be challenging as the accuracy depends on the cancelation of large matrix elements (as we discuss later). Fortunately, it turns out that the tanh function model enables us to analytically treat the transmission and reflection of propagating waves as well as properties of evanescent waves. Therefore, the analytical results can provide us with a precise description of imaging and absorption in these novel systems.

Before we systematically investigate the imaging properties of this structure, we confirm the existence of the large absorption near the zero-index point according to Ref. 6. The maximum absorption and several asymptotic properties are also addressed. We then analytically and numerically answer the question posted above about the effects of large absorption (arising from the zero-index point) on the perfect imaging. The main feature is that the image plane contains multiple subwavelength images of an object and the distance between the subimages is proportional to the characteristic transition length. This is a unique feature attributed to the gradient index of the perfect lens.

We note that this study provides an example of transition materials^9^,^10^,^11^,^12^,^13^ that can be treated fully analytically even for oblique incidence.

## Results

### Material model and its analytical solutions

**Figure 1:**
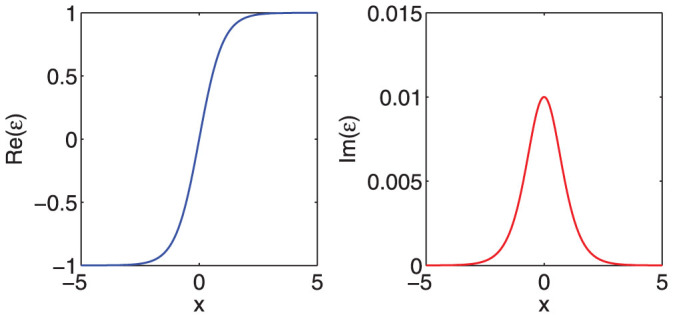
The permittivity *ϵ* of the material. *ρ* = 1, *δ* = 0.01.

The permittivity and permeability we use to model a gradient-index passing from positive to negative index are 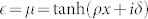. We note that 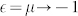 for 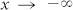 and 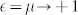 for 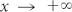. The parameter *ρ* characterizes the gradient of the transition. The imaginary part of 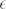 and *μ* is positive for 0 < *δ* < *π*/2, and therefore the material is a passive absorbing material under that condition. One example is shown in Figure 1. For simplicity, we set the velocity of light *c* = 1 and the vacuum parameters 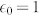 and *μ*_0_ = 1 in the whole study. As we treat all quantities as dimensionless, the results can be applied to different length scales after some rescaling.

The Helmholtz equation for TE waves have already been written as in [6], 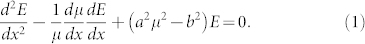
where *a* = *ω*/*ρ*, *b* = *ω* sin*θ*/*ρ* with *ω* and *θ* being the frequency and angle of the incident wave. This equation can be transformed into hypergeometric equation after changes of variables, *y* = *μ*^2^ and *E* = (1 − *y*)*^λ^F*(*y*) where 
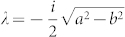
. The transformed equation is 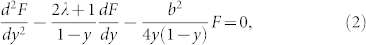
which is a hypergeometric equation with indexes *α* = *λ* + *ia*/2, *β* = *λ* − *ia*/2 and *γ* = 0.

### Analytical solutions for this material

In the following Methods section, we show all required steps to obtain the final analytical results. These steps generally involve the properties of hypergeometric equations and series. Here we only refer to the final results which are summarized in Eq.24.

### Basic properties of this material

In this part, we study some basic properties of the gradient-index material by investigating the analytical solutions. It includes the discussions about maximum total absorption rate and asymptotic behaviors for some limiting cases. These properties are helpful for us to understand the effects on imaging.

#### Maximum total absorption

As discovered in Ref. 6, the total absorption of this positive-to-negative transition material is nonzero even when the absorption coefficient is near zero. In this section, we confirm and develop this result for our specific model.

Before we investigate the total absorption in the lossless limit *δ* → 0, we first prove the identity 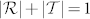, or equivalently 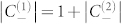. We can easily see that 
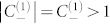
, since *α* and *β* are purely imaginary. Then we calculate 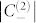. 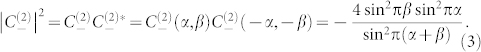
Then follows the identity 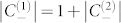.

Given the above identity, the total absorption 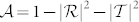 reaches its maximum 1/2 under the condition 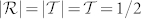. Then we finally arrive at the incident angle for maximum absorption, which is given by 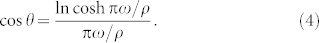
That is to say, even in the lossless limit *δ* → 0, for any given frequency, this material can present maximum total absorption 1/2 at a specified angle given by the above equation.

#### 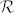 and 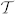 in several limiting cases

We only consider the limit *δ* → 0 in this section and do not introduce frequency dispersion to our model.

For oblique incidence *θ* ≠ 0 and high frequency 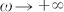, it can be easily proved that 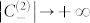. Therefore 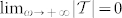, and 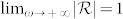. That is, for high frequencies, the light is totally reflected. However, this is the case when no dispersion is taken into consideration.

For oblique *θ* ≠ 0 but low frequency *ω* → 0, we have *α* → 0 and *β* → 0, followed by 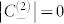, 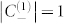 and 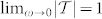, 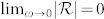. For low frequencies, the material behaves like a complementary material with total transmission.

For normal *θ* = 0 and a finite *ω*, we can easily get 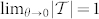, 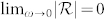. That means for normal incidence, we have total transmission which is the characteristic property of complementary materials suggested by Pendry and co-workers.

For large incidence angle near *π*/2 and a nonzero *ω*, we can prove that 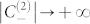. Then follows 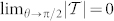, and 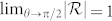. For glancing incidence, the light is totally reflected.

We should remind ourselves that for a sharp interface between the vacuum and negative-index material without smooth transition, the light will always be totally transmitted. We can see that for the gradient index, not only can we have a transmittance less than unity, but also have a nonzero reflectivity. Besides, the total absorption is nonzero even in the lossless limit *δ* → 0 as discussed in the previous section.

### Comparison with numerical calculations

**Figure 2:**
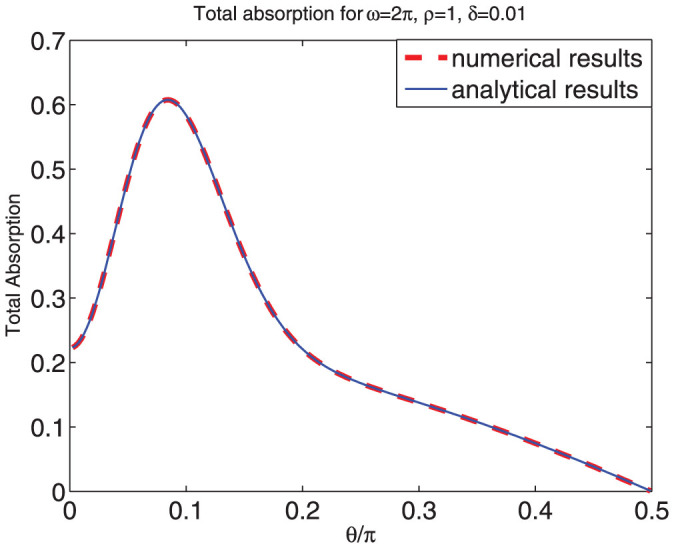
The comparison of the analytical and numerical results for the total absorption under *ω* = 2*π*, *ρ* = 1, and *δ* = 0.01. The numerical results are obtained by use of transformation matrix technique. The material is confined within [−5, +5], and is discretized into 3000 uniform layers.

In order to verify the analytical formula, we numerically compute the total absorption (
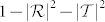
) of this material by use of the transfer matrix technique. The analytical and numerical results are compared in Figure 2 under the condition that *ω* = 2*π*, *ρ* = 1, and *δ* = 0.01. We can see that the two different methods give the same results. The total absorption can exceed the 1/2 bound because in this case we consider a finite absorption parameter *δ* = 0.01.

For evanescent waves, the numerical method based on the standard transfer matrix is inaccurate, because the nonzero transmission, which is necessary for perfect imaging, relies on the accurate cancellation of very large matrix elements. Therefore, if we want to accurately study the whole spectrum, analytical is the best tool.

### Effects on perfect imaging

In Ref. 1, Pendry proposed that a slab (perfect lens) with 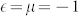 can overcome the diffraction limit. This is made possible by the fact that the perfect lens can ideally restore the information contained in the evanescent waves. However, for gradient-index material, the reflection and transmission can be very different from the sharp-interface case as we showed in previous sections. In the following, we analyze the effects of a graded transition from positive to negative index.

In the sharp-interface limit, 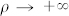, the transition materials approaches a half-infinite perfect-lens slab. A perfect image of an object in another side of the interface is therefore possible since the transmission for all transverse wave vectors are unity.

**Figure 3:**
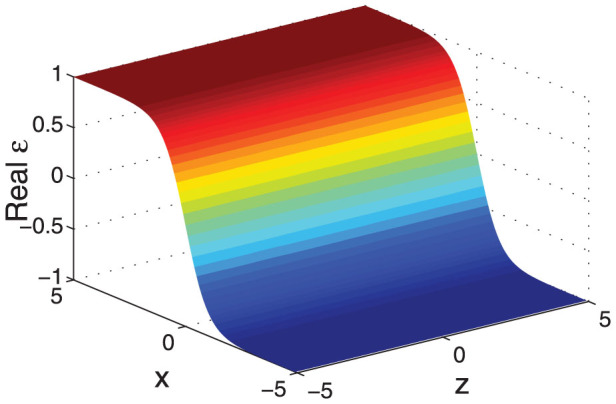
The setting of the material.

However, as can be seen from previous sections, if the transition is smooth (as in Fig. 3), i.e., *ρ* is finite, the transmission is not unity even for the propagating wave. If we set a monochromatic line object (not a point) in the plane *x* = −*a*, with an arbitrary transverse amplitude profile *E*(*x* = −*a*; *z*) and Fourier components 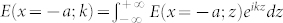, we will obtain the field amplitudes in the image plane *x* = *a*, 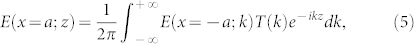
where, according to the results in Methods section, 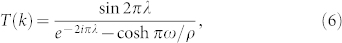
with 
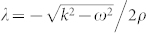
 being the principal value of the square root. We have also assumed that *δ* → 0.

Because *T* is a periodic function of *λ*, we can obtain the discrete Fourier expansion of *T*, 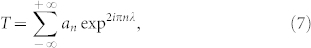
with 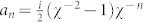 for 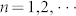; 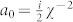; 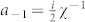; *a_n_* = 0 for 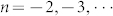. Here *χ* = cosh(*πω*/*ρ*).

A remarkable feature of each Fourier term 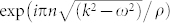 is that for large *k*, it approaches a periodic function and thus contributes a resonance when inversely Fourier-transformed. Let us approximate 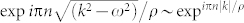 for large *k*. Then the images in the real *z* axis can easily be obtained after inverse Fourier transformation. 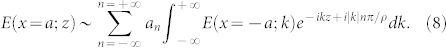
If we consider a simple Lorentzian object 
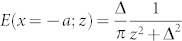
, the image should be 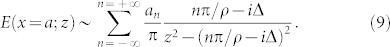
The above result indicates that the image plane contains multiple peaks. The widths of the all peaks remain the same as that of the object which is a Lorentz spot. The distance tween subimages is *π*/*ρ* which means that the emergence of multiple images can be attributed to a finite *ρ* or the gradient index. The amplitude *a_n_* of high order images exponentially decays with a characteristic order 
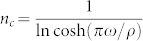
.

**Figure 4:**
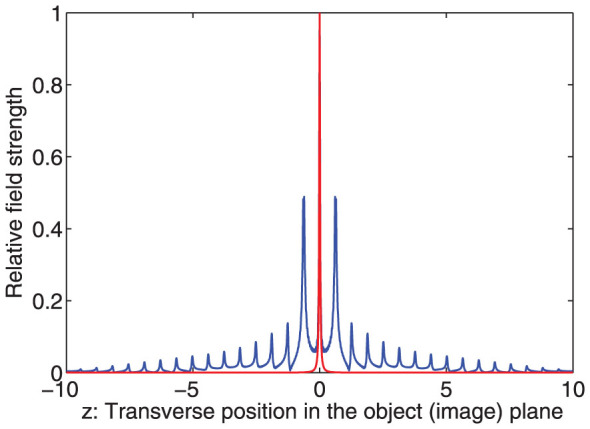
Single line. The field amplitude distribution in the object plane (red) and image plane (blue). The relative strength between the red and blue curve does not have meaning.

In order to obtain more accurate results for the image profile, we numerically integrate Eq.5 for the parameters *δ* = 0, Δ = 0.02, *ρ* = 5, *ω* = 1. These parameters means that the characteristic width of the transition layer is about 1/30 of the wavelength. The width of the line object is about 1/300 of the wavelength. The numerical results are displayed in Figure 4. That is to say, the width of the line object is much less than that transition width of the layer which itself is still much less than the wavelength. We can see that image profile appears in such a way that many peak emerges just as sub images of the line object. We can also verify that the distances, about *π*/5, between these peaks can be predicted by the Eq. 9 quite well. The widths of these sub images are comparable to that of the line object, which is also in accordance to the Eq. 9.

**Figure 5:**
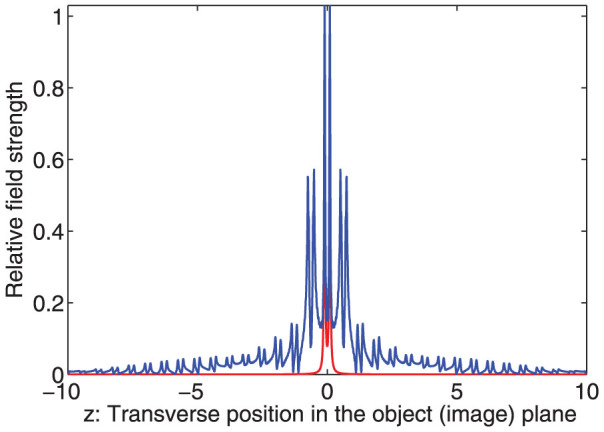
Image profile for a source of double line. The field amplitude distribution in the object plane (red) and image plane (blue). The relative strength between the red and blue curve does not have meaning. We use *d* = 10Δ, and other parameters are the same as in Fig. 4.

Let us study a more complex object, 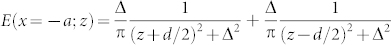, which contains double Lorentzian objects. Following the same procedure, we obtain the corresponding image 
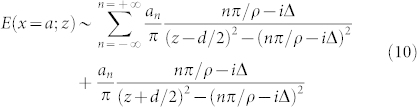
We can see that two sets of multiple images overlap with each other. We can confirm the approximation in Eq.(10) by direct numerical calculation, as shown in Fig. 5.

## Discussion

We have not included the finite-length effect (finite-length slab) in this study, and thus can not address the frequency cut off proposed in Ref. 3. If finite length, gradient index and absorption are all considered, we can expect that we still see multiple images but the detail profile of each subimage needs to be investigated. Since the material we discuss in this study is free of dispersion, we cannot address the formation of the image in the time domain. To analyze the more realistic material with dispersion we may consult to numerical methods and full analytical results are not possible. However, we can expect that it may take a relatively long time to form the image because of the field enhancement near the zero-index. These are to be studied in the future.

In summary, the transmission and reflection properties of a graded positive-to-negative materials can be very different from the ones with idealized sharp interface. These differences are mostly attributed to the large field enhancement near the zero-index point. To study the effects of graded index on perfect imaging, analytical solutions are obtained for smooth transition material with a “tanh” profile. The analytical expressions provide us insights into this problem. Expressing the fields in terms of hypergeometric functions, we obtain the closed-form analytical expressions for the reflection and transmission coefficients. The results are confirmed by direct numerical method. Our analytical results uncover the smooth transition effects on imaging: emerging of multiple images. The distance of these subimages is shown to be directly related to the gradient of the transition. These effects can be important for understanding the subtle properties of the perfect lens.

## Methods

### Solutions of the Helmholtz equation for 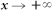, 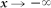 and 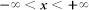


According to the material model we have adopted, for 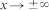, we have *μ* → ±1 and *y* → 1. As shown in the following section about Kummer solutions, the field can be expressed as linear superposition of two basic solutions, 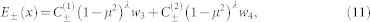
where *w*_3,4_, as well as *w*_1,2_ below, are Kummer solutions for hypergeometric equations (shown below).

For 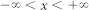, *y* is near the zero pole. The two basic solutions are, 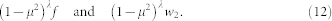
where *w*_2_, defined in Eq. 26, is one of the Kummer solutions near the zero pole and *f* is the solution with logarithm since we are working with a degenerate hypergeometric equation with index *γ* = 0.

Similar to the expression of Neumann functions in terms of Bessel function, the logarithm solution *f* can be expressed as limit of linear superposition of *w*_1_ and *w*_2_ of nonzero *γ* index, 
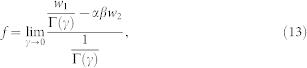
which will be proved in the Methods section. Specifically, according to the L'Hospital rule, 
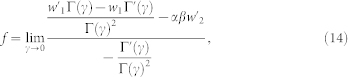
where 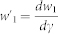, 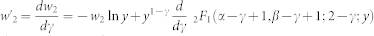 with _2_*F*_1_ being the hypergeometric function.

However, if we use the principal values of *f*(*y*), the field is discontinuous^14^ along the real *x* axis at zero point *x* = 0, corresponding to *μ* = tanh(*iδ*) = *i* tanh *δ*. Therefore, for 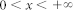 and 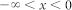, we should use different superpositions of *w*_2_ and *f* to make sure that the field is continuous.

The discontinuity of the principal value of *f* is *f* (*x* = 0+) − *f* (*x* = 0−) = 2*αβπiw*_2_(*x* = 0) according to Eq.(14). Then, if the field for 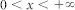 is 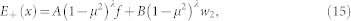
the field in 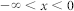 should be 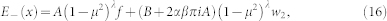
to ensure the continuity across *x* = 0.

#### Relations among 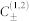 and A, B

The different field expressions (11, 15,16) should be equal in equivalent regions, which imposes connection relations among the coefficients 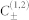 and *A*, *B*.

If we set 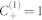, 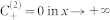 region, according to the Kummer connection formula between *w*_3_ and *w*_1,2_ in Appendix A, as well as Eq. (13,15), we have 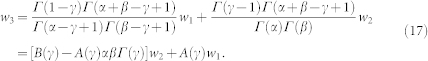
with *B* = lim*_γ_*_→0_*B*(*γ*),*A* = lim*_γ_*_→0_*A*(*γ*). After straightforward calculation, we have 
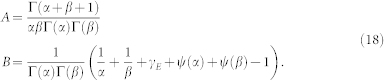
where *γ_E_* = −*ψ*(1) = 0.577215664…. and *ψ* is the derivative of the logarithm of Γ function.

Then according to Eq.(15,16,17), in 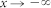


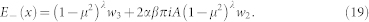
By use of Kummer connection formula between *w*_2_ and *w*_3,4_ in Appendix A, we have 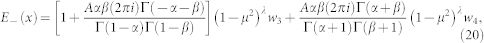
which, compared with Eq.(16), gives 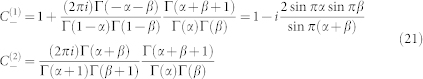


#### Reflection and transmission coefficients

Now we have obtained continuous solutions along the real *x* axis. For 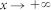, *E*_+_ = (1 − *μ*^2^)*^λ^w*_3_; for 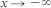, 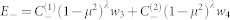. According to Appendix A, the asymptotic behaviors for 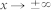 or *y* ≈ 1 are that *w*_3_ ≈ 1 and *w*_4_ ≈ (1 − *y*)^−2*λ*^.

Therefore, for 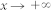, 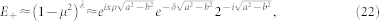
which is an outgoing wave; for 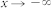, 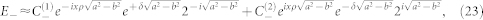
where 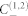 term represents incident and reflected wave, respectively, since the energy flux and wave vector point in opposite directions in this double negative region.

From Eq.(22,23) above, we can directly read out the reflection 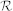 and transmission 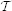 coefficients, 
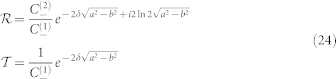


### Kummer solutions for hypergeometric equations

For completeness, we include some information about hypergeometric equations used in this study. One can also refer to mathematics handbooks. The hypergeometric equation is 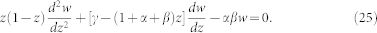
When none of *γ*, *γ* − *α* − *β*, *α* − *β* is an integer, the two basic solutions near 0 < |*z*| < 1 are, 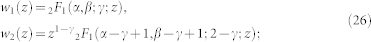
the two basic solutions for 0 < |*z* − 1| < 1 are, 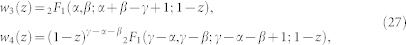
where 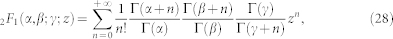
and definitions of these solutions on the whole complex plane are not shown here.

They are connected by Kummer connection formulas in common converging regions, which include 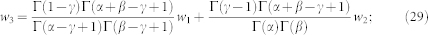

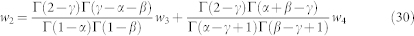


### Logarithmic solution near *z* = 0 for *γ* → 0

To prove Eq.(13), we only need to guarantee that its numerator 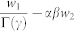 approaches zero when *γ* → 0.

According to Eq.(26), when *γ* → 0, 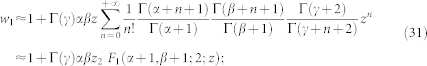
and 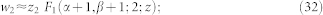
Then the Eq.(13) can be understood.

## Author Contributions

Y.D. performed the analytical and numerical calculations, drew figures and prepared the manuscript. C.T.C. proposed the research direction, supervised the work and revised the manuscript. R.P.W. reviewed the work.
